# Epidemiology and risk factors of hypoglycemia in subjects with type 1 diabetes in Brazil: a cross-sectional, multicenter study

**DOI:** 10.20945/2359-3997000000523

**Published:** 2022-10-03

**Authors:** Alexandre Barbosa Câmara de Souza, Maria Lúcia Cardillo Correa-Giannella, Marilia Brito Gomes, Carlos Antonio Negrato, Marcia Nery

**Affiliations:** 1 Universidade de São Paulo Faculdade de Medicina Departamento de Endocrinologia e Metabolismo São Paulo SP Brasil Departamento de Endocrinologia e Metabolismo, Faculdade de Medicina, Universidade de São Paulo, Hospital das Clínicas, São Paulo, SP, Brasil; 2 Universidade de São Paulo Hospital das Clínicas (LIM-18) Laboratório de Carboidratos e Radioimunoensaios São Paulo SP Brasil Laboratório de Carboidratos e Radioimunoensaios (LIM-18), Universidade de São Paulo, Hospital das Clínicas, São Paulo, SP, Brasil; 3 Universidade do Estado do Rio de Janeiro Unidade de Diabetes Departamento de Medicina Interna Rio de Janeiro RJ Brasil Departamento de Medicina Interna, Unidade de Diabetes, Universidade do Estado do Rio de Janeiro, Rio de Janeiro, RJ, Brasil; 4 Universidade de São Paulo Faculdade de Odontologia Programa de Doutorado em Medicina Bauru SP Brasil Programa de Doutorado em Medicina, Faculdade de Odontologia, Universidade de São Paulo, Bauru, SP, Brasil

**Keywords:** Type 1 diabetes, hypoglycemia, HbA1c target, alcohol abuse, social support

## Abstract

**Objective:**

The aim of this study was to investigate the factors associated with hypoglycemia and severe hypoglycemia (SH) in individuals with type 1 diabetes mellitus (T1D) in Brazil.

**Materials and methods:**

This multicenter, cross-sectional study was conducted between August 2011 and August 2014 across 10 Brazilian cities. The data were obtained from 1,760 individuals with T1D. Sociodemographic and clinical characteristics related to hypoglycemic events in the previous 4 weeks were evaluated.

**Results:**

Of 1,760 individuals evaluated, 1,319 (74.9%) reported at least one episode of hypoglycemia in the previous 4 weeks. The main factors associated with hypoglycemia were lower hemoglobin A1c (HbA1c) level, better adherence to self-monitoring of blood glucose (SMBG), and higher education level. Episodes of SH were reported by 251 (19%) individuals who, compared with subjects with nonsevere hypoglycemia, received lower doses of prandial insulin and higher doses of basal insulin, in addition to reporting less frequent use of long-acting basal insulin analogs. The percentage of SH episodes was evenly distributed across all ranges of HbA1c levels, and there were no correlations between the mean number of nonsevere or severe hypoglycemic events and HbA1c values. Higher alcohol consumption and more frequent hospitalizations were independently associated with SH.

**Conclusion:**

Although individuals presenting with hypoglycemia had lower HbA1c values than those not presenting hypoglycemia, there were no correlations between the number of nonsevere hypoglycemia or SH and HbA1c values. Also, the frequency of SH was evenly distributed across all ranges of HbA1c values. Better adherence to SMBG and higher education level were associated with hypoglycemia, while alcohol consumption, higher doses of basal insulin, and more frequent hospitalizations in the previous year were associated with SH.

## INTRODUCTION

The maintenance of adequate blood glucose (BG) levels in individuals with type 1 diabetes mellitus (T1D) delays the onset and progression of diabetic complications (1). Hypoglycemia is the most common adverse event associated with insulin therapy in these individuals and one of the major limiting factors for the achievement of glycemic goals, since the intensification of insulin therapy for attainment of lower HbA1c levels leads to an increased number of hypoglycemic events (2). The Diabetes Control and Complications Trial (DCCT) has found that strict glycemic control results in a three-fold increase in the number of hypoglycemic events in individuals with T1D (3).

Severe hypoglycemia (SH), defined as an event requiring third-party assistance, occurs in approximately 30% of the individuals with T1D and is an important cause of morbidity (4). Despite the vast literature about the pathophysiological mechanisms of diabetes-related hypoglycemia, real‐world data on the clinical incidence and predisposing clinical and social factors associated with this complication are scant, especially in developing countries (5,6).

A Brazilian study evaluating the association between hypoglycemia severity and some chronic complications of diabetes in individuals with T1D has shown heart rate variability, chronic kidney disease, and macrovascular complications to be independent predictors of moderate hypoglycemia and SH (7). Still, data on this topic are still lacking in our population (8).

Based on these considerations, the Brazilian Type 1 Diabetes Study Group (BRAZDiab1SG) performed a nationwide survey to evaluate the relationship between diabetes features and demographic and socioeconomic characteristics with the prevalence of nonsevere hypoglycemia and SH.

## MATERIALS AND METHODS

### Participants and study design

This was a cross-sectional, multicenter study conducted between August 2011 and August 2014 in 14 public secondary and tertiary health care clinics in 10 Brazilian cities located across all five main geographic regions of the country. The study design and methods have been previously detailed (6).

The sample included 1,760 individuals aged ≥ 13 years who had T1D and were following up at each center for at least 6 months. All subjects had been diagnosed between 1960 and 2014, had health care coverage from the Brazilian Unified Health Care System (SUS), and were under the care of an endocrinologist. The diagnosis of T1D was established by a physician based on the presence of typical clinical presentation of T1D, including variable degrees of hyperglycemia, weight loss, polyuria, polydipsia, polyphagia, and need for uninterrupted insulin use since diagnosis (9).

The study was conducted according to the principles of the Declaration of Helsinki and the Guidelines for Good Pharmacoepidemiology Practices. The local ethics committees at each center approved the protocol of the study, which followed international guidelines for human research.

### Data collection

The following variables were assessed using a questionnaire during clinical visits: current age, age at diagnosis, diabetes duration, height (meters), weight (kg), diabetes-related comorbidities, presence of diabetes complications (cardiac/cerebrovascular events, lower limb complications, retinopathy, diabetic kidney disease, sensorimotor neuropathy, cardiovascular autonomic neuropathy [CAN]), frequency of self-monitoring of blood glucose (SMBG), type of prescribed insulin therapeutic regimen (ITR), self-reported frequency of hypoglycemia in the previous month, and hospitalization due to any cause in the previous year (10).

Eligible participants were questioned about demographic and socioeconomic characteristics. Economic status was defined according to the Brazilian Economic Classification Criteria, with the following categories considered in the analysis: high, middle, low, or very low (11).

Evaluation of adherence to ITR was based on self-reported scales measuring the medication-taking behavior in the previous month, using the four questions proposed by Morisky (12).

The survey investigated the fasting levels of the following laboratory variables measured during the previous visit: plasma glucose (FPG) and serum hemoglobin A1c (HbA1c), total cholesterol, LDL-cholesterol, HDL-cholesterol, and triglycerides. The results were obtained from the participants’ medical records. Levels of HbA1c were measured using high-performance liquid chromatography (HPLC; Bio-Rad Laboratories, Hercules, California, USA). Subjects’ body mass index (BMI) was determined by dividing weight (kg) by squared height (m^2^).

### Definitions of hypoglycemia

Subjects with BG levels ≤ 70 mg/dL or those who required third-party help to recover from hypoglycemia during the previous month were included in the hypoglycemia group (HG).

Hypoglycemia was categorized as follows:

- SH, when the event required assistance by another person to actively administer carbohydrate, glucagon, or other resuscitative actions.
- Nonsevere hypoglycemia (NSH), when the event was managed by the individual alone (13).

Subjects who did not report any hypoglycemic episode in the previous 4 weeks were included in the non-hypoglycemia group (NHG) (14).

### Statistical analysis

Continuous variables are presented as mean ± standard deviation or median (25th-75th percentile) values, and discrete variables are presented as numbers (percentages). The participants were stratified into two groups according to hypoglycemia status, namely, NHG and HG. Those in the HG were further stratified into the SH and NSH groups. Comparisons of independent continuous variables were performed between categories of hypoglycemia using chi-square analyses and Student’s *t* test. Linear regression was used to assess the relationship between hypoglycemia status and HbA1c level. Quadratic regression provided the best fit for data distribution.

Factors associated with NSH (yes/no) and SH (yes/no) were examined using univariate and multivariate logistic regression, and those with a p value < 0.05 in the univariate analysis were included in the multivariate analysis. Odds ratios (ORs) and respective 95% confidence intervals (CIs) were computed in these analyses. P values < 0.05 were considered significant. All statistical analyses were performed using STATA 15.0 (StataCorp, College Station, Texas, USA).

## RESULTS

### Characteristics of the study population

The study included 1,760 subjects with T1D (48.3% men) with a mean age of 30.0 ± 11.9 years, mean diabetes duration of 14.5 ± 9.3 years, and mean HbA1c level of 9.0 ± 2.1%. In all, 55.5% participants had some microvascular complication. Table 1 shows the characteristics of the participants as a whole and grouped according to hypoglycemia status.

**Table 1:** Characteristics of the study population as a whole (“overall”) and categorized according to hypoglycemic status

Variable		Overall	Non-hypoglycemic group	Hypoglycemic group	P values
Number of subjects		1,760	441 (25.1%)	1,319 (74.9%)	
Sex (male)		771 (43.8%)	200 (45.4%)	571 (43.3%)	0.45
Age (years)		30.0 ± 11.9	28.2 ± 11.6	30.6 ± 12	0.93
Diabetes duration (years)		14.5 ± 9.3	13.6 ± 8.6	16.1 ± 0.94	<0.01
HbA1c (%)		9.00 ± 2.10	9.65 ± 2.55	8.80 ± 1.90	<0.01
Body mass index (kg/m^2^)		24.2 ± 4.2	24.1 ± 4.1	24.2 ± 4.2	0.63
Weekly alcohol consumption (Units*/week)		1.06 ± 1.12	1.05 ± 0.58	1.06 ± 1.23	0.12
Any physical activity during the week		441 (25.1%)	215 (48.7%)	226 (51.2)	0.80
Antidepressants use		137 (7.8%)	22 (5.0%)	115 (8.7%)	0.01
Monthly average family income (in Brazilian reais)		2,624.0 ± 2,260.6	2,351.4 ± 2,086.2	2,711.3 ± 2,307.6	<0.01
Access to supplementary health care		533 (30.3%)	104 (23.6%)	429 (32.5%)	<0.01
Average formal education (years)		12.2 ± 3.8	11.4 ± 9.2	12.5 ± 3.8	<0.01
Total daily insulin dose (IU/kg)		0.86 ± 0.38	0.89 ± 0.42	0.85 ± 0.37	0.03
Prandial insulin dose (IU/day)		18.8 ± 12.1	19.2 ± 11.9	18.7 ± 12.1	<0.01
Basal insulin dose (IU/kg)		0.59 ± 0.28	0.62 ± 0.31	0.58 ± 0.27	0.45
Continuous subcutaneous insulin infusion		62 (3.52%)	9 (2.04%)	53 (4.02%)	<0.01
Rapid-acting prandial insulin analog		876 (54.4%)	182 (46.4%)	760 (56.9%)	<0.01
Long-acting basal insulin analog		685 (46.4%)	127 (34.3%)	558 (50.4%)	<0.01
Self-monitoring of blood glucose		1,663 (94.5%)	392 (88.9%)	1,271 (96.4%)	<0.01
Adherence score		1.07 ± 0.97	0.99 ± 0.98	1.09 ± 0.96	0.12
Ketoacidosis at diabetes onset		453 (25.9%)	89 (20.2%)	364 (25.9%)	<0.01
Ketoacidosis in the previous year		54 (3.07%)	15 (3.40%)	39 (2.96%)	0.64
Hospitalizations in the previous year (per 100 patients/year)		27.0	24.5	28.0	0.39
Microvascular complications		976 (55.5%)	241 (54.6%)	735 (55.8%)	0.66
	Retinopathy	590 (35.9%)	125 (30.9%)	465 (37.5%)	0.02
	eGFR (mL/min)**	87.1 ± 30.1	91.8 ± 31.9	85.5 ± 29.3	<0.01
	Diabetic kidney disease	435 (25.2%)	96 (22.1%)	339 (26.3%)	0.09
	Sensorimotor neuropathy	307 (17.8%)	68 (15.6%)	239 (18.5%)	0.16
	CAN***	628 (36.4%)	170 (39.3%)	458 (35.5%)	0.15

Data are presented as number (percentage) or mean ± standard deviation.

* One alcohol unit = 8 g of pure alcohol.

** eGFR, estimated glomerular filtration rate calculated using the Chronic Kidney Disease Epidemiology Collaboration equation.

*** CAN, cardiovascular autonomic neuropathy.

### Features related to hypoglycemia

Of the 1,760 subjects evaluated, 1,319 (74.9%) reported at least one episode of hypoglycemia in the previous month. The characteristics of both groups (NHG and HG) are shown in Table 1. Subjects in the HG lived mostly in the Southeast region of Brazil and had longer diabetes duration, lower HbA1c values, higher monthly average family income, and higher education level.

Regarding diabetes treatments, subjects in the HG received a lower total dose of daily insulin and prandial insulin than subjects in the NHG. Use of continuous subcutaneous insulin infusion (CSII) and rapid-acting and long-acting basal insulin analogs were more frequent in the HG. Additionally, the frequency of SMBG was higher in the HG compared with the NHG.

The following diabetes-related complications were more frequent in the HG than the NHG: ketoacidosis at diabetes onset, retinopathy, and lower estimated glomerular filtration rate (eGFR). The overall presence of microvascular complications did not differ between groups, while more frequent use of antidepressants was observed in the HG.

Among the subjects in the HG, 1,068 (81%) had NSH and 251 (19%) had at least one episode of SH in the previous month. At least one episode of nocturnal hypoglycemia was reported by 320 (24.3%) subjects, while asymptomatic hypoglycemia was reported by 504 (38.8%) subjects. The subjects with asymptomatic hypoglycemia had a higher prevalence of CAN than those with SH (39.1% *versus* 33.2%, respectively, p = 0.03).

### Features related to severe hypoglycemia

Table 2 shows the characteristics of the participants grouped according to the severity of hypoglycemia. Compared with subjects in the NSH group, those in the SH group had slightly higher HbA1c values, lower BMI values, higher weekly alcohol consumption, lower monthly average family income, and less access to supplementary health care.

**Table 2:** Characteristics of the study population categorized according to the severity of hypoglycemia

Variable		Only nonsevere hypoglycemia	At least one episode of severe hypoglycemia	P values
Number of subjects		1,068	251	
Sex (male)		102 (40.6%)	468 (43.9%)	0.35
Age (years)		30.7 ± 12.2	30.2 ± 11.1	0.53
Age at diagnosis (years)		14.9 ± 8.8	13.6 ± 9.0	0.03
Diabetes duration (years)		15.9 ± 9.4	16.8 ± 9.2	0.18
HbA1c (%)		8.76 ± 1.90	8.97 ± 2.03	<0.01
Body mass index (kg/m^2^)		24.3 ± 4.2	23.6 ± 3.9	<0.01
Any physical activity during the week		554 (51.9%)	130 (52.0%)	0.80
Alcohol consumption (/Units*/week)		0.944 ± 0.58	1.81 ± 2.87	<0.01
Use of antidepressants		23 (9.2%)	92 (8.6%)	0.78
Monthly average family income (in Brazilian reais)		2,805.9 ± 2,382.5	2,307.7 ± 1,908.6	<0.01
Access to supplementary health care		363 (34%)	66 (26.3%)	0.02
Average formal education (years)		12.2 ± 3.9	12.2 ± 3.7	0.21
Daily insulin dose (IU/kg)		0.84 ± 0.35	0.86 ± 0.41	0.47
Prandial insulin dose (IU/day)		19.1 ± 12.2	17.1 ± 11.6	0.02
Basal insulin dose (IU/kg/day)		0.57 ± 0.26	0.61 ± 0.32	0.04
Rapid-acting prandial insulin analog		573 (58.2%)	121 (51.7%)	0.09
Long-acting basal insulin analog		474 (52.5%)	84 (41.2%)	<0.01
Continuous subcutaneous insulin infusion		48 (4.49%)	5 (1.99%)	0.09
Self-monitoring of blood glucose		1,024 (95.9%)	247 (98.4%)	0.16
Adherence score		1.08 ± 0.96	1.19 ± 1.00	0.191
Ketoacidosis in the previous year		26 (2.4%)	13 (5.2%)	0.02
Hospitalizations in the previous year (per 100 patients/year)		22	53	<0.01
Microvascular complications		567 (53.2%)	168 (66.9%)	<0.01
	Retinopathy	352 (35.0%)	113 (48.1%)	<0.01
	Diabetic kidney disease	258 (24.7%)	81 (32.9%)	<0.01
	eGFR (mL/min)**	85.6 ± 28.8	84.9 ± 31.5	0.729
	Sensorimotor neuropathy	183 (17.5%)	56 (22.9%)	0.05
	CAN***	353 (33.7%)	105 (42.9%)	<0.01

Data are presented as number (percentage) or mean ± standard deviation.

* One alcohol unit = 8 g of pure alcohol.

** eGFR, estimated glomerular filtration rate calculated using the Chronic Kidney Disease Epidemiology Collaboration equation.

*** CAN, cardiovascular autonomic neuropathy.

Regarding diabetes treatment, subjects in the SH group versus those in the NSH group received lower doses of prandial insulin and higher doses of basal insulin. The use of long-acting basal insulin analogs was less frequent in the SH group.

The following diabetes-related complications were more frequent in the SH group than the NSH group: ketoacidosis in the previous year, retinopathy, diabetic kidney disease, CAN, and overall presence of microvascular complications. Hospitalizations in the previous year were also more frequent in the SH group.

**Figure 1:**
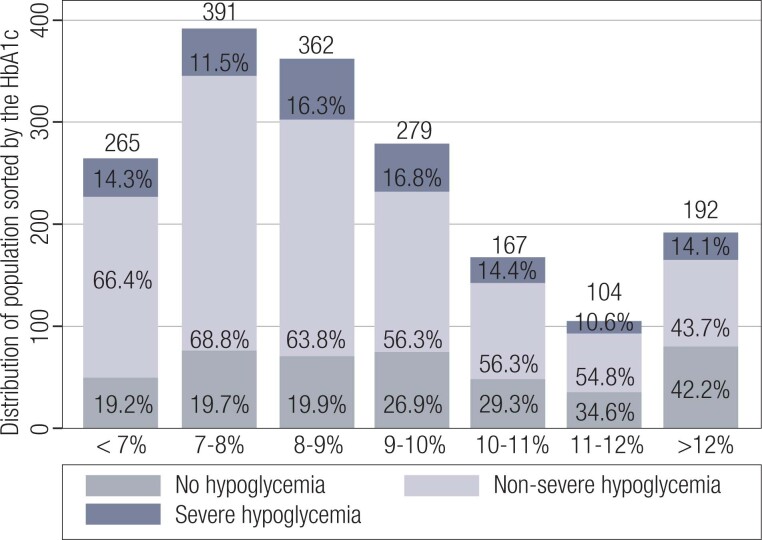
Relationship between ranges of hemoglobin A1c (HbA1c) values and percentages of hypoglycemic events.

The percentage of SH events was evenly distributed across all ranges of HbA1c values (Figure 1). No correlations were observed between the mean number of nonsevere (r = 0.02; p = 0.42) or severe (r = 0.12; p = 0.08) hypoglycemic events and HbA1c values.

Adjusted multivariate analysis with SH as the dependent variable revealed that the variables independently associated with this condition were higher weekly alcohol consumption and hospitalizations in the previous year (Table 3).

**Table 3:** Final adjusted logistic regression analysis with severe hypoglycemia as the dependent variable

Independent variables	Odds ratio (95% CI)	P value
Basal insulin dose	0.30 (0.01-7.32)	0.46
Prandial insulin dose	0.99 (0.93-1.05)	0.76
Long-acting basal insulin	0.51 (0.11-2.25)	0.37
Age at diabetes diagnosis	0.96 (0.89-1.04)	0.36
Access to supplementary health care	1.11 (0.20-6.28)	0.90
Monthly average family income	1.00 (1.00-1.00)	0.75
Weekly alcohol consumption	5.64 (1.45-21.94)	0.01
Retinopathy	0.60 (0.08-4.79)	0.63
Diabetic kidney disease	0.68 (0.10-4.67)	0.69
CAN*	5.38 (0.76-38.06)	0.09
Microvascular complications	0.65 (0.05-8.16)	0.74
Hospitalizations in the previous year	2.01 (1.03-3.93)	0.04
Body mass index	0.93 (0.82-1.06)	0.30

* CAN, cardiovascular autonomic neuropathy.

## DISCUSSION

In this large, population-based, observational study, NSH and SH in the previous 4 weeks occurred in, respectively, 74.9% and 19.0% of the subjects with T1D. Similar results have been reported in the HYPOS-1 study, which found a prevalence of 78.6% of hypoglycemic events (15). Our cohort had a lower incidence of SH than the cohort in the DCCT trial, in which the prevalence of this complication was reported at 68% in the intervention group and 35% in the control group. We have no explanation for this finding, although it is possible that, compared with the participants in our cohort, those in the DCCT trial may have had more access to supplementary health care and a higher education level, which may explain greater awareness about hypoglycemic episodes in that cohort (16).

Surprisingly, we found no correlations between the mean number of NSH or SH episodes and HbA1c values. Additionally, the percentage of SH episodes was evenly distributed across all ranges of HbA1c values. Although some clinical trials have shown that intensive glucose control strategies increase the risk of hypoglycemia (17), the results of most recent studies have reported conflicting results in terms of an inverse correlation between HbA1c values and hypoglycemia. A reanalysis of the DCCT trial has shown that achieving a mean HbA1c level of ~7% had increased by roughly 12% the risk of SH compared with a mean HbA1c level of 9% (18). Similar results have been presented by Haynes and cols. (19) and Redondo and cols. (20), who found a nonlinear association between hypoglycemia and HbA1c levels. Additionally, HbA1c values < 7% have not been associated with higher rates of hypoglycemia compared with HbA1c values between 8%-9%, indicating that the HbA1c value is a minor predictor of SH (21).

Despite having lower HbA1c values, individuals in the HG had a higher frequency of retinopathy and lower eGFR values, which are probably related to a longer diabetes duration in this group and poor glycemic control in the first years after T1D diagnosis (22).

The higher frequency of CSII use in the HG was probably due to reverse causality, since an excessive number of hypoglycemic events is among the indications for this therapeutic modality. The higher frequency of SMBG observed in the HG probably reflects the concern and fear of hypoglycemia by these individuals, which in turn, may increase concerns related to health and explain the more frequent use of antidepressant medications in this group in the univariate analysis (7,23). Although depression may lead to psychobiological changes, such as dysregulation of the autonomic nervous system, higher cortisol levels, and increased inflammatory markers potentially worsening glycemic control, an association between the use of antidepressants and hypoglycemia was not observed in the multivariate analysis (7).

The association between higher family income and increased frequency of hypoglycemia reinforces the data reported in previous studies suggesting that a worse financial status leads to less access to health resources, including glucometers and test strips, not allowing the diagnosis of mild hypoglycemic episodes (24,25).

An interesting finding of the present study was the lack of difference in insulin doses between individuals with and without SH. However, the group with SH received a higher dose of basal insulin, lower dose of prandial insulin, and fewer long-acting basal insulin analogs, which are features that could be implicated in the increased frequency of SH (26-28).

The subjects in the SH group had worse socioeconomic status than those in the NSH group. A lower average family income leads to less access to supplementary health care, limiting treatment options even when the subject receives care through the public health system. The underutilization of medications due to high costs may worsen the individual’s glycemic control, leading endocrinologists to increase the doses of some medications, which may prompt hypoglycemia during intermittent use of such medications (29-31). Additionally, better adherence to SMBG and higher education level seem to improve the awareness of hypoglycemic episodes (32,33).

Previous data have implicated alcohol in up to one-fifth of hospital admissions due to hypoglycemia in individuals treated with insulin (34,35). Alcohol intake may lead to hypoglycemia through different mechanisms, as it disrupts the individual’s ability to perceive hypoglycemia by directly impairing hormonal counterregulatory responses to low BG levels (35-37). Additionally, alcohol intake increases cognitive deficits associated with hypoglycemia in subjects with T1D (34,38).

The present study has several limitations. Because of its cross-sectional design, conclusions about causality cannot be drawn; thus, our understanding of the observed associations is limited. In addition, our sample does not represent the Brazilian population since all included individuals lived in large cities, received medical care from public health care centers and were treated by specialists. Thus, subjects who rely on primary care facilities and live in rural areas were not represented. We also used only clinical criteria to define T1D. We did not measure autoantibodies against beta cells or C peptide levels, which could have led us to misclassify some cases as having T1D (9,10). However, clinical criteria defining T1D are frequently used in studies with large samples like the present one (18,39). There is also the possibility of survivorship bias since subjects with a history of more frequent SH events could have had an earlier death (30).

In conclusion, although subjects in the HG had a significantly lower mean HbA1c value than those in the NHG, no correlations were observed between the number of NSH or SH episodes and HbA1c values. Also, the frequency of SH was evenly distributed across all ranges of HbA1c values. Better adherence to SMBG and higher education level were associated with the occurrence of hypoglycemia, while alcohol consumption and more frequent hospitalizations in the previous year were associated with the occurrence of SH. The main messages of the present study are the importance of avoiding high doses of basal insulin and educating individuals about alcohol consumption and its consequence on glycemic control, a simple measure that can contribute to reducing SH events.
